# Two-stage exchange of infected total hip arthroplasty with a dual-mobility cup is associated with a low instability rate

**DOI:** 10.1051/sicotj/2025013

**Published:** 2025-03-20

**Authors:** Nicolas Zadel, Céline Cazorla, Anne Carricajo, Thomas Neri, Frédéric Farizon, Bertrand Boyer

**Affiliations:** 1 Chirurgie Orthopédique et Traumatologie, Centre Hospitalier du Forez Montbrison 42600 France; 2 Centre de Référence des Infections Ostéo-Articulaires complexes (CRIOAc) de Saint Etienne Saint-Étienne 42270 France; 3 Maladies Infectieuses et Tropicales, CHU de Saint Etienne Saint Etienne France; Univ Jean Monnet, INSERM, CIC1408, FCRIN, I-REIVAC, RENARCI, ANRS 42270 France; 4 Infectious Agents and Hygiene department, CHU de Saint Etienne Saint Etienne France; Univ Jean Monnet, CIRI, Centre International de Recherche en Infectiologie, GIMAP team, Université de Lyon, INSERM, U1111, CNRS, UMR5308, ENS Lyon, Université Claude Bernard Lyon 1 Lyon 69008 France; 5 Chirurgie Orthopédique et Traumatologie, CHU de Saint-Etienne 42270 France; 6 Univ Jean Monnet, INSERM, Mines Saint-Étienne, U1059 SAINBIOSE Saint-Étienne 42270 France

**Keywords:** Prosthetic joint infection, Hip, Dual mobility, Dislocation, Two-stage

## Abstract

*Introduction*: The two-stage management of hip Prosthetic Joint Infection (PJI) is faced with a high rate of dislocation. Dual mobility (DM) cups have proved effective in reducing the risk of dislocation, but few data are available on the two-stage management of hip PJI. The objectives of this retrospective cohort study were to analyze the rate of dislocation, and the rate of recurrent dislocation and to identify risk factors for dislocation. Our hypothesis was that the use of a DM cup during a two-stage replacement had a low instability rate. *Methods*: Data from 70 two-stage changes with DM cup reimplantation performed in our centre between 2011 and 2020 were retrospectively collated. The mean age was 69 years [18–93], with a mean follow-up of 3.4 years [1.5–9.6]. Dislocation rates and risk factors for prosthetic instability were collected. Univariate and multivariate analyses were performed to identify risk factors favouring prosthetic instability. *Results*: The rate of dislocation at the last follow-up was 8.6% (6/70), including 4.3% (3/70) in patients with no infection recurrence. The rate of recurrent dislocation was 0% when infection was controlled. The occurrence of spacer dislocation, the presence of immunosuppressive and antiaggregant medication, the local grade of the McPherson score and infection treatment failure were associated with the occurrence of a dislocation. No risk factors were identified in the multivariate analysis. *Discussion*: Compared with the rates reported in the literature, the use of a DM cup seems indicated in this context in order to lower the risk of recurrent dislocation. Preventing spacer dislocation and infection recurrence seems to be essential to avoid the risk of instability of the future prosthetic hip.

## Introduction

Prosthetic Joint Infection (PJI) is a rare but formidable complication [1]. Dale et al. [2] reported a 0.6% revision rate from the Nordic register.

Since single-stage replacement has numerous theoretical contraindications, two-stage replacement is still considered the reference treatment [3]. This major surgery is still a source of complications with death rates ranging from 0% to 26% [4] and a re-intervention rate of 20% [5]. Dislocation, especially recurrent prosthetic dislocation or instability, accounts for a significant proportion of these complications. The infectious process that could damage capsular healing, muscular atrophy of the abductor muscles and repeated operations leading to bone loss and loss of functionality could explain the rate of dislocation, which varies between 10% and 15% in the series reported [5–7]. This figure may be underestimated [8].

Furthermore, instability is one of the main reasons for revision of primary total arthroplasties, with a rate of 15–25% [6]; it is even the leading cause of revision in the United States, accounting for 22% of all revisions while in France the rate is lower, in fifth place accounting for 10.4% of revisions [9]. One reason for the low rate of instability in France could be the high proportion of Dual Mobility (DM) Cups, as these implants were developed to prevent the risk of dislocation, both in primary total hip arthroplasties (THRs) [10] and revision THRs [11, 12].

To our knowledge, no study has specifically investigated the rate of dislocation of a DM cup during the two-stage management of a chronic infection in a total hip prosthesis. Our hypothesis was that the use of a DM cup in this septic context would have a low risk of postoperative instability.

We, therefore, conducted a retrospective study to assess the rate of prosthetic dislocation (isolated and recurrent dislocation) of a DM cup after the insertion of a spacer following a chronic infection in a total hip prosthesis and to assess possible risk factors for prosthetic instability.

## Methods

After obtaining the approval of the institution’s ethics committee, retrospective data were collected from our centre’s prospective database. Between January 2011 and October 2020, all patients requiring explantation of a total hip were reviewed. Exclusion criteria were no reimplantation, follow-up of less than 18 months, reimplantation with a standard, non-DM cup and a history of an initial two-stage management for prosthesis infection during the study period.

**Figure 1:**
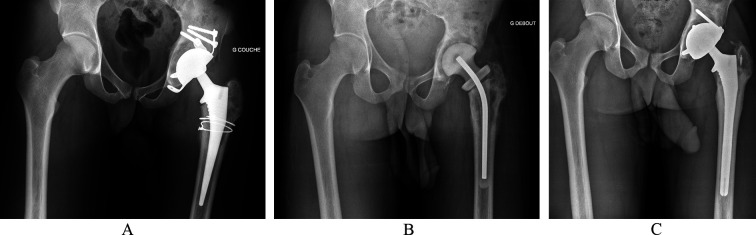
First case of Hip chronic Prosthetic Joint Infection. Male, 18 years-old, history of THA for avascular necrosis (Sickle-cell disease) 6 months ago. Pathogen: Methicillin-sensitive *Staphylococcus aureus*. (A) Pre-operative AP X-ray. (B) Preformed articulated spacer. (C) 4-year follow-up AP X-ray.

**Figure 2:**
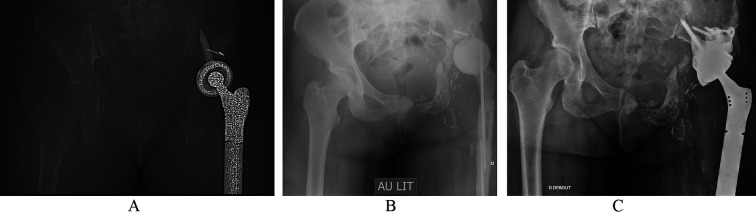
Second case of Hip chronic PJI. Female, history of osteosarcoma of the acetabulum 11 years ago. Pathogen: *Streptococcus agalactiae*. (A) Pre-operative AP X-ray. (B) Preformed articulated spacer. (C) 2-year follow-up X-ray.

The diagnosis of THR infection was made in accordance with the criteria of the Musculoskeletal Infection Society [3]. Prosthetic removal surgery was indicated in cases of chronic infection [13] (Figures 1A and 2A).

A total of 70 hips out of 70 patients were included. The characteristics of the study population are summarised in Table 1. McPherson classification [14] was used to stratify the PJI according to local and systemic criteria.

**Table 1:** Population.

Variables	Overall size *N* = 70
Age	69.2 ± 14.4 [18–93]
Gender M (%)/F (%)	36 (51.4)/34 (48.6)
BMI	28.4 ± 6.4 [17–43]
ASA score	
1	5 (7.1)
2	31 (44.3)
3	28 (40)
4	6 (8.6)
McPherson stage	
IIIA1	3 (4)
IIIA2	11 (14.7)
IIIA3	3 (4)
IIIB1	11 (14.7)
IIIB2	24 (32)
IIIB3	6 (8)
IIIC1	9 (12)
IIIC2	2 (2.7)
IIB2	1 (1.3)

BMI: Body Mass Index; ASA: American Society of Anesthesiology.

The first surgical stage was performed via a posterolateral approach with the removal of all prosthetic and ancillary hardware. A thorough synovectomy was performed with pulsed lavage and placement of an antibiotic spacer (Figures 1B and 2B, OneStage^TM^, Zimmer-Biomet, Warsaw, USA from 2011 to 2015, then Subiton^TM^, Prothys Orthopédie, Brive-la-gaillarde, France, from 2015 to 2020). Antibiotic therapy was delivered for a minimum of 6 weeks. The decision to perform the second stage was validated by the complex Bone and Joint Infections Reference Centre [15, 16] on clinical and biological parameters of infection control after antibiotics had been discontinued for at least 2 weeks. The second stage of surgery was performed via a posterolateral approach. Thorough washing with pulse lavage was performed, and samples were taken. The level of implant stabilization was planned pre-operatively on the basis of the radiographic assessment and reassessed intraoperatively. An additional femoral osteosynthesis or bone allograft was performed depending on the intraoperative findings. Antibiotic therapy was reintroduced as soon as the second set of samples was completed and was subsequently reassessed on the basis of the bacteriological results. Regular follow-up was instituted for clinical, biological and radiographic monitoring (Figures 1C and 2C).

Dual Mobility Cups used were all third-generation DMCs from SERF (Décines, France), either uncemented (Novae-E TH^®^, Novae COPTOS TH^®^) or cemented in a cage (Novae STICK^®^).

The primary endpoint was the rate of dislocation at the last follow-up, defined as early dislocation before the third-month post-op. We also assessed the rate of recurrent dislocation, which is defined as the second or subsequent recurrence. The patient’s condition and data relating to the pre- and post-explant surgical context were also collected in order to identify risk factors for dislocation.

Patient characteristics included age at reimplantation, gender, Body Mass Index (BMI), American Society of Anesthesiology (ASA) score, presence of immunosuppressive treatment (anti-metabolites, corticoids, chemotherapies, immunotherapies, monoclonal antibodies) or treatment modifying haemostasis, smoking, alcohol consumption, presence of chronic inflammatory arthropathy, diabetes or neuromyopathy.

The pre-explantation surgical context included radiographic analysis (localized periosteal appositions and radiolucent lines according to Gruen et al. [17] or O’Neill and Harris [18] and cementation of the acetabulum with the location of radiological signs according to the DeLee and Charnley zones [19]), number of previous hip operations, history of dislocation, C-Reactive Protein (CRP) level, indication and approach of initial arthroplasty, condition of greater trochanter, cementing of implants, type of implants, lower limb length inequality, time from onset of clinical signs to first stage surgery, and time from initial arthroplasty to explantation.

The postoperative surgical context included the duration of the second operation, the method of prosthetic extraction, complications related to the spacer, the time between the first and second operation, the type of implant and the use of cement during the second operation, the presence of pathogens during the second operation, the condition of the greater trochanter and the presence of calcifications at the last follow-up (Brooker classification [20]), the positioning of the acetabulum and the change in offset at the last follow-up, the occurrence of a septic recurrence.

Statistical analyses were performed using SPSS software (SPSS Inc, Chicago, USA). There were no missing data. Continuous variables are presented as mean ± standard deviation [min–max]. Fisher’s exact test was used for categorical variables. The Student’s *t-*test and the Mann-Whitney test were used to compare continuous variables following a normal or non-normal distribution, respectively. Binomial logistic regression was used to analyze the influence of patient background and surgical characteristics on the occurrence of dislocation. All tests were two-tailed. The significance level was set at 0.05 with a 95% confidence interval.

## Results

The mean follow-up was 3.4 ± 2.8 years [1.5–9.6]. Seventeen per cent of patients had died at the last follow-up (12 patients). The mean age at death was 81.4 ± 5.9 years [72–89]. No patient died from a recurrence of infection on the reimplanted prosthetic hip. The mean time from reimplantation to death was 3.5 ± 2.2 years [1.1–7.5].

Over the whole cohort, 10 dislocations were recorded in 6 patients (6 hips, 8.6%). The median time to first dislocation was 44 days, with 4 early dislocations and 2 late dislocations. Of these 6 patients, 4 had only one episode. Another one of these patients had a single recurrence. The last patient presented a first episode at 6 months post-implantation, requiring bloody reduction, then iterative dislocations (4 episodes) concomitant with the impossibility of controlling the infection. Arthroplasty resection (Girdlestone) a year and a half after reimplantation was necessary to control the infection. There were no other cases of recurrent dislocation, the rate being 1.4% (1/70) in the cohort as a whole and 16.6% (1/6) in the subgroup of unstable patients. The mean follow-up time for patients with dislocation was 4.4 ± 2.4 years [1.7–9.6].

The presence of immunosuppressive or antiaggregant therapy was statistically associated with the occurrence of postoperative dislocation. Thirty-eight percent (3/8) of patients on immunosuppressive therapy developed a dislocation, compared with 5% (3/62) of immunocompetent patients (*p* = 0.034) (Table 2). Twenty-one percent (4/19) of patients on antiaggregants developed dislocation compared with 4% (2/51) of other patients (*p* = 0.042).

**Table 2:** Variables.

Variables	Stable hips	Unstable hips	Significance
Antiaggregants			
Yes	15	4	*p* = 0.042
No	49	2	
Immunosuppressants			
Yes	5	3	*p* = 0.017
No	59	3	
McPherson Local score			
1	10	4	*p* = 0.030
2	43	2	
3	11	0	
Spacer dislocation			
Yes	6	3	*p* = 0.025
No	58	3	
Infection recurrence			
Yes	7	3	*p* = 0.034
No	57	3	

The rate of dislocation was statistically different between the different local grades of the McPherson score. Twenty-nine percent (4/14) of patients stratified as grade 1 had a dislocation compared with 4% (2/45) and 0% (0/11) respectively in grades 2 and 3 (*p* = 0.030).

None of the variables relating to the surgical context preceding the first stage was associated with the occurrence of post-implantation dislocation.

The occurrence of spacer dislocation was statistically associated with the occurrence of postoperative dislocation in the second stage, with 30% (3/9) of patients with spacer dislocation developing prosthetic dislocation, compared with 5% (3/61) of patients without spacer dislocation (*p* = 0.025) (Table 2).

The rate of dislocation was higher in patients with septic recurrence, with 30% (3/10) of patients with septic recurrence developing post-reimplantation dislocation compared with 5% (3/60) of patients without septic recurrence (*p* = 0.034).

The multivariate model did not identify any risk factor independently associated with the risk of post-reimplantation dislocation.

## Discussion

Instability after two-stage management for hip infection remains a major problem.

We report a rate of dislocation at last follow-up of 8.6% (6/70).

In their series of two-stage exchanges for PTH infection, which did not specifically include DM cups, Hartman and Garvin [7], McAlister et al. [5], and Petis et al. [6] reported dislocation rates of 14.7%, 10.5%, and 12% respectively. These rates appear higher than those found in our series.

The rate of recurrent dislocation was 1.4% (1/70). In the series by McAlister et al. [5], 58% of unstable patients had at least one recurrence, and 79% of these patients required revision for instability. In the series by Petis et al. [6], 54% of unstable patients underwent at least one revision surgery for instability. These rates appear to be much higher than those reported in our series, with 17% of patients (1/6) undergoing revision surgery for a mixed cause (persistent instability and inability to control infection).

The previously cited series did not find septic recurrence to be a risk factor for post-reimplantation dislocation. In our series, excluding septic recurrence, the rate of dislocation was 5% (3/60), and the rate of recurrent dislocation was 0% (0/60). The septic context of our cohort probably contributes to an overestimation of the risk of dislocation.

In our series, the occurrence of spacer dislocation, the local McPherson score, the occurrence of septic recurrence and the presence of immunosuppressive and antiaggregant treatment were statistically associated with the occurrence of post-implantation dislocation in the univariate analysis. The multivariate model did not, however, reveal any independent dislocation risk factor.

The risk factors for dislocation in two-stage septic hip reimplantation have received little attention in the literature [5]. Garceau et al. [21], and Chalmers et al. [22] also found an association between spacer dislocation and the occurrence of post-implantation dislocation. This result can be explained firstly by the fact that the risk factors leading to spacer dislocation are the same as those contributing to the instability of a prosthetic hip. Secondly, the insufficient superior-external acetabular coverage provoked by the impaction of an unstable spacer could also explain this result [23]. There is no published series that specifically addresses the problem of dislocation in one-stage DM cup revision. The subgroup analysis of the series of one-stage changes by Abdelaziz et al. [24] found the use of a DM cup to be a factor in reducing postoperative dislocation. The absence of an intermediate stage with a spacer to reduce the risk of dislocation, inherent in two-stage management, could increase the benefit of DM cups in one-stage management.

In our series, the local grading of the McPherson score was correlated with a risk of postoperative dislocation. Further studies would be needed to test the hypothesis of scar tissue influencing dislocation, for example, by assessing stiffness in greater depth.

The presence of chronic inflammatory arthropathy is recognized as a risk factor for dislocation in primary arthroplasties [9, 25]. In our series, 50% of patients with chronic inflammatory arthropathy were on immunosuppressive therapy for an arthropathy-related disease. This variable could potentially be a confounding factor, as multivariate analysis did not find this factor.

In our series, the rate of dislocation in patients without septic recurrence was 5% compared with 30% in patients with septic recurrence. The persistence of infection probably contributes to the risk of dislocation and could explain our dislocation rate of 8.6% for all patients combined.

The main limitation of our study is a lack of statistical power on a small number of patients.

Instability remains a major problem in the two-stage management of chronic hip infection and is one of the leading causes of revision surgery. The use of a DM cup reduces the risk of postoperative dislocation and recurrent dislocation. The occurrence of spacer dislocation or recurrent infection was correlated with the risk of post-implantation instability. Preventing and detecting these complications seems to us to be an essential point in reducing the risk of instability of the future prosthetic hip.

## Data Availability

Blinded data from this study are available on request.
